# Examining the Impacts of the Coronavirus Pandemic and Social Distancing on the Health of People With Mobility Disabilities

**DOI:** 10.3389/fpubh.2022.875880

**Published:** 2022-04-27

**Authors:** Kelsey Shinnick Goddard, Jonathan Schulz, Isaac Nzuki, Jean P. Hall

**Affiliations:** ^1^Research and Training Center on Independent Living, University of Kansas, Lawrence, KS, United States; ^2^Vermont Center on Behavior and Health, Larner College of Medicine, University of Vermont, Burlington, VT, United States

**Keywords:** coronavirus, disability, health, pandemic, social distancing

## Abstract

**Objective:**

To understand the effects of the coronavirus pandemic on the health and participation of people with mobility disabilities living in the community.

**Methods:**

Participants responded to a survey designed to assess the effects of the coronavirus pandemic on their health and access to health care. Participants identified if various life situations were worsened, unchanged, or improved during the pandemic. Participants could provide further information on their improved or worsened lived experience in open-ended questions.

**Results:**

A total of 39 people with mobility disabilities responded to the survey. Results indicate that many experienced a worsening of life situations related to health, including access to medications, health care services, and transportation.

**Conclusions:**

Results show that many experiences were caused by the lack of appropriate policies, rather than the pandemic itself. Therefore, there is a need to modify pandemic preparedness plans and other policies to meet the needs of people with disabilities.

## Introduction

The coronavirus outbreak was formally named as a pandemic by the World Health Organization in the spring of 2020 (1). Although the pandemic continues to have an impact on all people living in the United States, research shows that people with disabilities are disproportionately affected by the pandemic and the subsequent social distancing orders across the country (2). Despite this fact, the experiences of people with disabilities are regularly disregarded by public health experts and policymakers (3). The disproportional effects on health and health care that people with disabilities are experiencing may very well represent the most prominent and least publicly recognized crisis that Americans are facing at this time (4).

For people with disabilities who rely on social supports for daily care needs, social distancing was never a realistic choice. Even so, social distancing mandates and widespread panic resulted in many community-dwelling people with disabilities going without necessary care. Throughout the United States, reports document instances of personal care assistants abandoning people with paralysis in their homes, often leaving them unable to get out of their beds (5). This situation has literally translated into people with disabilities going days without bathing, toileting, or eating, which can lead to adverse effects on health, such as the development of pressure sores, urinary tract infections, and malnutrition. In addition to the physical effects on health, social abandonment, and isolation can have numerous effects on the mental health of people with disabilities, including psychological well-being (6) and increased anxiety, depression, and suicidal ideation (7).

To provide context to these events, independent living and deinstitutionalization movements have sought to shift care from institutional settings (e.g., nursing homes) to home- and community-based settings (8). Although these movements have provided people with disabilities opportunities for greater choice and control over the setting in which they live, the shift to community living has had the unintended consequence of siloing many of them during the coronavirus pandemic. For people receiving home- and community-based services under a Medicaid waiver, emergency “backup” plans are identified as a key component of person-centered planning (9). However, virtually no research has been conducted that identifies the effectiveness of these backup plans during a true emergency (10). Because of this lack of emergency preparedness, the coronavirus pandemic has often been devastating to some people with disabilities living in the community.

Specifically, some people with physical/mobility disabilities may be at increased risk for unmet social support and health care needs during the coronavirus pandemic due to their complex health needs, low socioeconomic status, and a lack of external social support (11). For some people with mobility disabilities, interruptions in public transportation alone may affect all facets of life if those services provide the only option for accessible and affordable travel (12). Additionally, many of the solutions provided to the community at large, such as pharmaceutical delivery, may not be financially feasible to someone who has a limited income (13). Given that some people with mobility disabilities are likely to have complex heath care needs, even these subtle changes to daily living activities may result in substantial impacts on health (14).

Unfortunately, if people with mobility disabilities did experience a health care need during a time of social distancing mandates, reports show that many health care professionals closed their doors to the public, making health care inaccessible (15). Although telemedicine was presented as a solution to many people opting to continue meeting with their medical providers using a remote format, people with disabilities report many barriers to receiving equitable levels of remote care (16). Reports also show that many health care facilities implemented practices to postpone “elective” surgeries or procedures to address the burden of hospital care (17). For people with mobility disabilities, however, these interruptions to community-based rehabilitation services have translated to increased recovery time or loss of function (18).

Detailed, firsthand accounts of the lived experiences of people with mobility disabilities as they relate to the pandemic are lacking (19). Although a growing literature base has focused on statistically documenting disparities in disease burden or outcomes, there has been comparatively little focus in the academic literature on the experiences of people with disabilities during the pandemic and how those experiences might inform public health policy and practice in the future. This paper is a timely contribution and can serve as a call for more work on the topic. Thus, the purpose of the present study is to explore and capture the impacts of the coronavirus pandemic on the lived experiences of people with mobility disabilities living in the community, particularly impacts on physical and mental health, access to everyday health needs (e.g., medical supplies, prescription medications), and access to health care (e.g., doctors, specialists, counselors).

## Methods

### Participants

We conducted this research within the context of the Research and Training Center on Promoting Interventions for Community Living (RTC/PICL), which includes a study designed to test the effectiveness of a multifaceted intervention in increasing the community participation of people with mobility disabilities. Although the presented sub-study to document the effects of the coronavirus pandemic was not the original focus of the larger RTC/PICL study, we felt a quick pivot was important to gain timely insight on pandemic-related effects for people with mobility disabilities. Thus, participants actively enrolled in the RTC/PICL study were asked if they would like to participate in a sub-study to document the experiences of people with mobility disabilities during the coronavirus pandemic. Inclusion criteria for participants enrolled in the original RTC/PICL included being 18 years or older, being one's own guardian, living in the community, and having a mobility disability, with or without other disabilities. To conduct this study and the larger RTC/PICL study, researchers partnered with Centers for Independent Living (CILs), which are community-based, nonresidential agencies that provide an array of advocacy and other services to people with disabilities. At the start of the coronavirus pandemic (April-June 2020), three of the participating CILs in the United States—located in Indiana, Ohio, and Pennsylvania—elected to recruit a sub-sample of RTC/PICL participants to participate in a survey to document the effects of the coronavirus pandemic on enrolled consumers. These three CILs recruited a sample of 39 consumers to participate. Participants received a $25 incentive payment for participating.

### Measures

Our team developed a survey to document the experiences of people with mobility disabilities during the coronavirus pandemic. Approximately half of these questions asked participants about effects related to community-based services and supports (e.g., personal assistance services, social relationships, grocery access), which are analyzed in a corresponding manuscript (20). The other set of questions focused on the health and access to health care of people with mobility disabilities, which is the focus of this manuscript. Specifically, participants responded to eight questions related to health, including “How has the coronavirus/social distancing affected your (a) access to medications, (b) access to transportation, (c) access to medical supplies, (d) access to medical providers; (e) access to health services, (f) access to mental health services, (g) mental health, and (h) physical health?” Examples of services were provided (e.g., examples of dental care, physical/occupational therapy, dialysis, and chemotherapy were provided for “health services”). Participants could respond that their access or condition had either remained unchanged, worsened, improved, or was not applicable. If participants selected “worsened” or “improved,” they then had the opportunity to describe how their condition had worsened or improved via an open-ended response option.

Finally, participants self-reported their frequency of in-person visits to health-related facilities (i.e., health care providers, pharmacies, exercise facilities) in the last 7 days. Participants had previously responded to this question in a pre-survey questionnaire during their participation in the larger RTC/PICL study prior to the coronavirus pandemic. Thus, the survey questions served as a post-measure for comparative analyses.

### Data Collection

After the CIL staff had informed the participants about the sub-study and confirmed participation interest, participants were asked if they would prefer to complete the survey online or over the phone. Participants who chose to complete the survey online were sent an electronic survey link via email. These participants provided electronic consent to participate and responded independently to all survey questions, including open-ended response options. Participants who chose to complete the survey over the phone were called by one of the research team members. These participants provided oral consent to participate, and responses were recorded by the research team. Open-ended responses were transcribed in real-time by the researcher during the call, and responses were read back to the participant to confirm the accuracy of recorded responses. All study procedures were approved by the Institutional Review Board (IRB) at the University of Kansas.

### Data Analysis

Descriptive statistics were calculated for demographics-related variables. For qualitative analysis, key themes were determined prior to analysis based on the open-ended response topics in the survey. Two research team members independently selected open-ended responses using an inductive approach to identify illustrative quotations relevant to each theme. These researchers met to compare selected quotations and to reach consensus on representative quotations to include for each theme. For quantitative analysis, a frequency analysis was conducted to consider the number of applicable responses indicating unchanged, worsened, or improved. Participants could elect to skip survey questions or to respond “not applicable” to any of the response topics, so total responses to each survey topic vary somewhat, as described in the results. Finally, the average number of visits to health-related facilities before and after the coronavirus pandemic were calculated.

## Results

### Demographics

Table 1 depicts demographic characteristics of participants. Results show that participants were a mean of 53.3 years of age and were mostly female (62%), White (80%), and unmarried (80%). Additionally, most respondents reported having at least some college education (71%) and an annual household income of ≤$20,000 (59%).

**Table 1:** Participant characteristics.

**Characteristic**	***n* (%)**
**Age**	
*M* = 53.28, *SD* = 16.41, Range = 24-92	
18-34	7 (17.9)
35-64	23 (59.0)
65+	9 (23.1)
**Gender**	
Male	15 (38.5)
Female	24 (61.5)
Race^*^	
American Indian/Alaskan Native	1 (2.6)
Black/African American	5 (12.8)
White	31 (79.5)
Other	3 (7.7)
Hispanic/Latino	1 (3)
Missing	1 (2.6)
**Marital status**	
Married	8 (20.5)
Separated, divorced, widowed	14 (35.9)
Never been married	13 (33.3)
Unmarried couple	4 (10.3)
**Education**	
Less than high school diploma	2 (5.3)
High school graduate	9 (23.7)
Less than bachelor's degree	13 (34.2)
Bachelor's degree	10 (26.3)
Master's degree or higher	4 (10.5)
Missing	1 (2.6)
**Household income**	
$10,000 or less	11 (28.2)
$10,001 to $20,000	12 (30.8)
$20,001 to $40,000	10 (25.6)
More than $40,000	6 (15.4)
**Employment status**	
Employed	12 (30.8)
Not employed	27 (69.2)
**Benefits**	
Supplemental security income	10 (25.6)
Social Security disability insurance	17 (43.6)
Social Security retirement	6 (15.4)
None	5 (12.8)
Other	9 (23.1)

*M, Mean; SD, Standard Deviation*.

* *No participants identified as Asian or Native Hawaiian/Pacific Islander*.

### Worsened, Unchanged, and Improved Life Situations

Table 2 depicts self-reported life situations that worsened, remained unchanged, or improved during the coronavirus pandemic. Representative quotations from participants who provided open-ended responses related to their improved or worsened experiences are included below.

**Table 2:** Self-reported worsened, unchanged, and improved life situations during the coronavirus pandemic.

**Themes**	**Worsened access (%)**	**Unchanged access (%)**	**Improved access (%)**
Access to medical providers (*n* = 38)	60.5	34.2	5.3
Access to health services (*n* = 35)	48.6	51.4	0.0
Access to transportation (*n* = 38)	42.1	55.3	2.6
Access to medical supplies (*n* = 34)	23.5	76.5	0.0
Access to medications (*n* = 37)	18.9	75.7	5.4
Access to mental health services (*n* = 19)	47.4	52.6	0.0
Physical health (*n* = 37)	40.5	56.8	2.7
Mental health (*n* = 37)	48.6	43.2	8.1

#### Access to Medical Providers

Participants with worsened access to medical providers (60.5%) described the effects of service policies made obsolete by the pandemic:

“*I have not been able to obtain an appointment for referral to a specialist until yesterday when they called me and stated they are now authorized to do new patient appointments over the phone. Until then, I had been unable to schedule the appointment with the urologist for over a month.”*

Participants also noted the effects of staff layoffs:

“*Reaching my primary care physician via phone has also been a challenge as they have laid off their entire staff except for one person.”*

In addition, participants noted the effects of limited appointment windows:

“*Appointments at my doctor's office are only happening during the morning, so getting an appointment, especially with my own doctor, is difficult to impossible.”*

Finally, participants noted the effects of office closures:

“*My three doctors were all down during the coronavirus. Two will still be down for the next month. Doctors and dentists were literally not accessible during the coronavirus. If there was an emergency, I would have to use the emergency room.”*

Participants who reported improved access (5.3%) did not elaborate on ways their access had improved.

#### Access to Health Services

Participants with worsened access to health services (48.6%) described the effects of the postponement of elective surgeries:

“*I am waiting for a hip replacement and have to wait for them to let the doctors do surgeries. And I am in pain but thank goodness for medications.”*

Participants also noted personal decisions to delay services due to perceived risk:

“*Physical therapy was prescribed, but not begun due to the coronavirus. Exercise facilities have been closed for a month. As a result, I am significantly less flexible and losing strength.”*

Finally, participants noted the effects of facility closures:

“*Everything is on hold. I have an abscess[ed] tooth right now, and [the dentist] told me to call back in a month. They put me on antibiotics, but that doesn't help the swelling and the pain.”*

#### Access to Transportation

Participants with worsened access to transportation (42.1%) described the effects of service disruptions:

“*I had to cancel my doctor's and dentist [appointments] and I am going to have to look for new providers that are in walking distance to my house… When I had transportation, [my providers] were always driving distance away. Now, I need to get other providers that are walking distance from my house.”*

Participants also noted effects of disruptions on access to prescribed medications:

“*[I have] difficulty getting to the pharmacy and no delivery available per my insurance.”*

Finally, one respondent who reported improved access noted that their paratransit service was able to provide increased schedule flexibility and reduced transportation costs:

“*Fewer people are riding paratransit, so they are more able to accommodate my schedule. Rides are free until the coronavirus threat subsides.”*

#### Access to Medical Supplies

Participants with worsened access to medical supplies (23.5%) described the barriers in obtaining prescriptions for supplies:

“*Because of COVID and minimal medical supply, [I] can't get [nebulizer] tubing… without a special prescription, and none of my doctors are open, they just tell me to go to the emergency room or keep trying.”*

Participants also noted effects of insurance-related delays:

“*I am having difficulty getting adjustments to my current wheelchair seating due to call centers being overwhelmed to check coverage with my insurance company.”*

In addition, participants noted effects of supply shortages:

“*Getting over-the-counter supplies like protein drink and rubbing alcohol, disinfectants, incontinence supplies is more difficult due to shortages.”*

Participants also noted their perceived risk to obtain supplies:

“*The DME provider loaning me equipment poses a potential risk [to my health].”*

Participants noted effects of changes to store return policies:

“*An elbow brace that I had bought at CVS ended up being too small and I was not allowed to return or exchange it due to their recent policy changes due to the risk from COVID-19.”*

Finally, participants noted effects of provider closures:

“*My wheelchair needs repairs, and I usually go in to have these done because my apartment is too small to do repairs in. However, the provider is not allowing the public in at this time, which is forcing me to wait.”*

#### Access to Medications

Participants with worsened access to medications (18.9%) described effects due to office closures:

“*[I had] difficulty getting changes to medications due to no appointment or referral appointments until recent updates in telephone appointments.”*

Participants also noted effects of prescription delays:

“*I have had to wait longer for physician renewal of prescriptions.”*

In addition, participants noted supply shortages:

“*[I have] difficulty in finding over-the-counter medications and supplies due to shortages of supply in stores and online shopping.”*

Participants noted the effects of telecommunication barriers:

“*[My doctor] is going to do a phone call about my [prescription], but wants to do a video call and my internet is not secure and it drops and stuff. [My doctor] said this time she will do a phone call, but next time if I need a refill or anything, it will need to be a video call or in person. If I can't do that, I won't get a refill and she may drop me.”*

Finally, participants who reported improved access suggested that the ability to receive prescriptions through the mail and refill prescriptions via telehealth services made accessing their medications easier:

“*I [used] to have to see the doctor every 90 days to get [my] prescription refilled… So now I can get the prescriptions without having to see the doctor, because there isn't anything wrong, just [my disability], so it's actually kind of improved.”*

#### Access to Mental Health Services

Participants with worsened access to mental health services (47.4%) described effects of provider policies to not accept new patients:

“*I was not currently receiving mental health services, but with my increased depression from all this social isolating I should be, but I don't know how to find anyone taking new patients right now.”*

Participants also noted concerns around family members or caregivers overhearing confidential information:

“*[My] therapist calls me every two weeks, and we do that over the phone… Hard to talk when everybody is in the house, and you've got to say personal things. I can't wait to go back into her office.”*

In addition, participants noted effects of telecommunication barriers:

“*I've had to do my counseling on video calls, and I have poor internet, so it's difficult.”*

Finally, participants noted anxiety about using telehealth for mental health services:

“*In order for my insurance to pay for mental health services, I have to be treated by a licensed therapist. All licensed therapists [are] not doing home visits… I don't anticipate much progress if I don't feel safe or comfortable talking through a webcam.”*

#### Physical Health

Participants with worsened physical health (40.5%) described the effects of inactivity:

“*My physical health has decreased because I cannot work out, and because of my knee I cannot walk. So basically, weight gain is an easy thing to do because there is nothing else to do.”*

Participants also described increased pain from inactivity:

“*Because I don't go out except in my own yard, the amount of my exercising has gone way down, and my mobility pain has risen.”*

Finally, one respondent who reported improved physical health noted the effects of increased time to focus:

“Since the isolation, I have time to focus on the things that are important in life.”

#### Mental Health

Participants with worsened mental health (48.6%) described effects of not leaving the home:

“*I am teetering on the depressed side right now, as I have not left my home in a month. My anxiety has been worrisome as well.”*

Participants also noted increased anxiety around the pandemic:

“*My anxiety is sky high as I'm afraid of getting COVID-19 and dying.”*

In addition, participants noted the effects of social isolation:

“*The isolation has become the hardest obstacle. I speak to friends, but I miss the physical interactions. Because of increased anxiety, I'm having a lot of difficulty sleeping and staying focused during the day.”*

Finally, participants who reported improved mental health (8.1%) described effects of slowed pace, increased time alone, and decreased social expectations:

“*It's actually improved my mental health because it's allowed me to take things in and properly assess things in my life [instead of] having my mind race a mile a minute.”*

### Frequency of Health-Related Facility Visits

Participants reported the number of in-person visits made to visit (a) a doctor or health care provider, (b) a pharmacy, and (c) an exercise facility, before the pandemic and during the survey period. Overall, results displayed in Table 3 show that participants reported decreased numbers of visits to all health-related facilities during the pandemic.

**Table 3:** Self-reported frequency of visits to health-related facilities before and during the coronavirus pandemic.

	**Average number of visits**	
**Variable**	**Pre-pandemic**	**During-pandemic**
How many times have you visited doctors or health care providers in the past 7 days? (*n* = 38)	1.50	0.18
How many times have you visited pharmacies in the past 7 days? (*n* = 37)	1.03	0.61
How many times have you visited exercise facilities in the past 7 days? (*n* = 38)	0.47	0.00

*Data were excluded if they did not have a matching pre- and post-test data point*.

## Discussion

Our results highlight the numerous adverse effects that the coronavirus pandemic has had on the health and access to health care of people with mobility disabilities. In every survey health topic area, at least some respondents reported worsened access or health during the pandemic. Collectively, these responses document the experiences of people with disabilities during a pandemic, which may help inform future emergency preparedness planning and response efforts. In general, our results highlight that many of the barriers experienced by people with disabilities were not due directly to the coronavirus infection, but rather to subsequent social policies and practices that may have been mitigated with effective emergency preparedness planning and response. In fact, previous research (21) has suggested that people with disabilities may be four times more likely to be injured or to die during disaster situations than people without disabilities “not because of their ‘vulnerable' position, but because urban health policy, planning, and practice have not considered their needs” (22).

First, our results highlight the need to quickly adapt existing health care policies to accommodate people with mobility disabilities. Participants cited numerous barriers due to inflexible policies, such as policies that intake appointments were not allowed to be conducted *via* telehealth, medications could not be prescribed over the phone, and stores could no longer accept medical supply returns or exchanges. These examples highlight the socially induced barriers that may be presented when policies remain inflexible to accommodate people with disabilities during a pandemic. Given that many people with disabilities are covered by Medicaid and/or Medicare, federal policies to require or allow providers to accept new patients *via* telehealth or make prescriptions over the phone would likely have been helpful and can still be considered now or in the future. Similarly, insurance policies disallowing coverage for prescription deliveries seem short-sighted and potentially cost-ineffective and should be flexible during times of social distancing mandates.

Second, our results highlight telecommunication barriers. Although many medical professionals provided telemedicine as a way to continue medical care, this format presents additional barriers for some people with disabilities (16). Research shows that people with disabilities are more likely to live in poverty and are less likely to have access to internet services (23). Among those with internet access, our respondents indicated that internet reliability and speed were both barriers to effective telehealth experiences. Additionally, certain services, such as physical, occupational, or speech therapy may be less effective or even impossible when not conducted in-person. Similarly, remote delivery of psychiatric services may be problematic when a family member or care attendant is consistently present in the home. As one of our participants noted, it is “hard to talk when everybody is in the house, and you've got to say personal things,” a finding also echoed by other researchers (24).

Third, our results highlight the reduced service capacity observed due to cited office closures, staff layoffs, and treatment postponements. Although these strategies were employed to reduce virus exposure risk through health care settings, our results show that these access delays may result in dangerous and immediate risk to people with disabilities who are unable to obtain necessary medical supplies and services. Among other issues, participants noted delays in getting a hip replacement, completing physical therapy, being able to access exercise facilities, getting repairs to wheelchairs, and getting emergency dental treatment. Thus, when developing emergency preparedness plans, it is imperative to also consider the severity of effects that unmet health care needs may have on people with disabilities. For example, policies may need to be put in place that make exceptions to which services are suspended, or for whom, so that people with disabilities still receive needed care.

Fourth, our results highlight barriers due to supply shortages. Our participants cited multiple difficulties in obtaining necessary medications and medical supplies. Although some shortages occurred due to manufacturer and supplier closures due to rising infection rates, reports show that many shortages occurred because people were “panic buying” for emergency or backup supplies and did not necessarily have a history of use (25). Thus, these shortages may have occurred due in part to inadequate policies in place to discourage hoarding or to reserve medications and supplies for people with disabilities who did have a history of use. To prepare for similar events in the future, emergency preparedness planners may need to reassess the supplies needed in their consumable medical supply list.

Fifth, our results highlight the poor health outcomes that may be observed during a pandemic, unrelated to the coronavirus itself. With respect to physical health, participants reported adverse effects due to inactivity, muscle atrophy, increased pain, and weight gain. Participants also reported adverse effects on mental health due to increased anxiety, prolonged time spent at home, and social isolation. Although it may be arguable that these effects are unavoidable due to the coronavirus pandemic and social distancing, it is possible that these effects may have been exacerbated due to underlying health conditions, inflexible health care policies, office closures, reduced service capacity, telecommunication barriers, and supply shortages.

Finally, it is worth noting that some respondents reported positive experiences related to the pandemic and social distancing policies. For example, some individuals appreciated the time and space for introspection and the opportunity to re-focus. One participant reported increased flexibility in not needing to visit the doctor before getting refills for a maintenance medication. Others appreciated the option for telehealth and fewer physical trips to the doctor. And, finally, another reported improved access to paratransit services, with more flexibility in scheduling and reduced costs due to the pandemic. These positive experiences can also be used to inform policy. For example, telehealth as an option reimbursable by insurance even post-pandemic may increase access to health care for some people with disabilities. Increased access to broadband and appropriate technology for people with disabilities would improve this option even more. Similarly, continued flexibility in scheduling and reduced costs for paratransit might increase not only access to health care for people with disabilities, but also to other forms of community participation.

These results contribute to the preliminary research needed to document the impact of the coronavirus pandemic on the health of people with mobility disabilities to better inform emergency preparedness planning and response now and in the future. Although this manuscript does not explore all possible solutions to address the identified impacts, it is imperative to first learn directly from the people whose lives were affected. Indeed, no policy affecting the lives of people with disabilities should be enacted without their representation. Thus, in order to move forward with identifying solutions, it is critical to first learn from the experiences of people with disabilities so that they can have a voice and authority to advocate for social policies that affect their lives.

### Limitations

There are a number of limitations in the present study. First, participants were a convenience sample of people with mobility disabilities from a small sample size, which included representation from only three different areas of the country. Therefore, the study may not accurately reflect the range of experiences people with varying disabilities throughout the United States experience in a pandemic. Additionally, the diversity of this sample is lacking in that the majority of our participants were White females. Second, participants did not elaborate on why their experiences remained unchanged. Therefore, information related to why a situation may have remained unchanged remains unknown. Finally, we developed the survey as a team in response to the ongoing pandemic, and therefore the survey was not assessed for validity or reliability. However, due to the novelty of the situation, it was necessary to develop our own survey in a short time period.

## Conclusions

The purpose of this study was to document the pandemic-related health experiences of people with mobility disabilities living in the community. Although all possible solutions are not elaborated upon here, this research highlights the importance of including people with disabilities in all levels of emergency planning and response (26). It is only through pausing to learn from the detailed, firsthand experiences of people with disabilities that policymakers, researchers, stakeholders, and disability advocates can learn how to adequately prepare and plan for their needs—so that we all may move forward *together*.

## Data Availability Statement

The raw data supporting the conclusions of this article will be made available by the authors, without undue reservation.

## Ethics Statement

The studies involving human participants were reviewed and approved by University of Kansas Institutional Review Board. The patients/participants provided their written informed consent to participate in this study.

## Author Contributions

KG and JH: study conception and design. KG, JS, and IN: data collection and analysis and interpretation of results. KG: draft manuscript preparation. All authors reviewed the results and approved the final version of the manuscript.

## Funding

The contents of this manuscript were developed under a grant from the National Institute on Disability, Independent Living, and Rehabilitation Research (NIDILRR grant number 90RT5043). NIDILRR is a Center within the Administration for Community Living (ACL), Department of Health and Human Services (HHS).

## Author Disclaimer

The contents of this manuscript do not necessarily represent the policy of NIDILRR, ACL, HHS, and you should not assume endorsement by the Federal Government.

## Conflict of Interest

The authors declare that the research was conducted in the absence of any commercial or financial relationships that could be construed as a potential conflict of interest.

## Publisher's Note

All claims expressed in this article are solely those of the authors and do not necessarily represent those of their affiliated organizations, or those of the publisher, the editors and the reviewers. Any product that may be evaluated in this article, or claim that may be made by its manufacturer, is not guaranteed or endorsed by the publisher.

## References

[1] World Health Organization. Director-General's Opening Remarks at the Media Briefing on COVID-19. Geneva: World Health Organization (2020).

[2] Linehan C Aranten-Bergman T Baumbusch J Beadle-Brown J Bigby C Birkbeck G. COVID-19 IDD: A global survey exploring the impact of COVID-19 on individuals with intellectual and developmental disabilities and their caregivers. HRB Open Res. (2020) 3:39. 10.12688/hrbopenres.13077.1 33392440 PMC7745183

[3] Vanderbom KA Eisenberg Y Tubbs AH Washington T Martinex AX Rauworth A. Changing the paradigm in public health and disability through a knowledge translation center. Int J Environ Res Public Health. (2018) 15:328. 10.3390/ijerph15020328 29438334 PMC5858397

[4] Guidry-Grimes L Savin K Stramondo JA Reynolds JM Tsaplina M Burke TB. Disability rights as a necessary framework for crisis standards of care and the future of health care. Hastings Center Rep. (2020) 50:28–32. 10.1002/hast.1128 32596899

[5] Sable-Smith B. People With Disabilities Find the Coronavirus Has Cut Them Off From Their Caregivers. Available online at: https://www.npr.org/sections/healthshots/2020/06/16/875944357/people-with-disabilities-find-the-coronavirus-has-cut-them-off-from-their-caregi (accessed June 16, 2020).

[6] Dalise S Tramonti F Armienti E Niccolini V Caniglia-Tenaglia M Morganti R. Psycho-social impact of social distancing and isolation due to the COVID-19 containment measures on patients with physical disabilities. Eur J Phys Rehabil Med. (2021) 57:158–65. 10.23736/S1973-9087.20.06535-1 33165314

[7] Mousavi SB. Coronavirus disease 2019 pandemic: Do not forget patients with severe mental illness. Int J Soc Psychiatry. (2020) 67:830–2. 10.1177/0020764020939982 32633183 PMC8559176

[8] The 2014 federal home and community-based services regulation: What you need to know. (2014). Available online at: https://thearc.org/wp-content/uploads/forchapters/NPM_HCBS_Final.pdf (accessed June 16, 2020).

[9] O'Keeffe J Saucier P Jackson B Cooper R Mckenney E Crisp S. Understanding Medicaid Home and Community Services: A Primer, 2010 Edition. Washington, DC: Department of Health and Human Services and RTI International (2010).

[10] Sabatello M Burke TB McDonald KE Appelbaum PS. Disability, ethics, and health care in the COVID-19 pandemic. Am J Public Health. (2020) 110:1523–7. 10.2105/AJPH.2020.305837 32816541 PMC7483109

[11] Epstein S Campanile J Cerilli C Gajwani P Varadaraj V Swenor BK. New obstacles and widening gaps: A qualitative study of the effects of the COVID-19 pandemic on U.S. adults with disabilities. Disabil Health J. (2021) 14:101103. 10.1016/j.dhjo.2021.101103 33840617 PMC9760300

[12] Mahmoudi E Meade MA. Disparities in access to health care among adults with physical disabilities: analysis of a representative national sample for a ten-year period. Disability Health J. (2015) 8:182–90. 10.1016/j.dhjo.2014.08.007 25263459

[13] Jesus TS Bhattacharjya S Papadimitriou C Bogdanova Y Bentley J Arango-Lasprilla JC. Lockdown-related disparities experienced by people with disabilities during the first wave of the COVID-19 pandemic: scoping review with thematic analysis. Int J Environ Res Public Health. (2021) 18:6178. 10.3390/ijerph18126178 34200979 PMC8228347

[14] Lund EM Forber-Pratt AJ Wilson C Mona LR. The COVID-19 pandemic, stress, and trauma in the disability community: A call to action. Rehabilit Psychol. (2020) 65:313. 10.1037/rep0000368 33119381

[15] Rubin R. COVID-19's crushing effects on medical practices, some of which might not survive. J Am Med Assoc. (2020) 324:321–3. 10.1001/jama.2020.11254 32556122

[16] Lebrasseur A Fortin-Bédard N Lettre J Bussières EL Best K Boucher N. Impact of COVID-19 on people with physical disabilities: A rapid review. Disabil Health J. (2021) 14:101014. 10.1016/j.dhjo.2020.101014 33158795 PMC7603994

[17] Annaswamy TM Verduzco-Gutierrez M Frieden L. Telemedicine barriers and challenges for persons with disabilities: COVID-19 and beyond. Disability Health J. (2020) 13:100973. 10.1016/j.dhjo.2020.100973 32703737 PMC7346769

[18] Al-Jabir A Kerwan A Nicola M Alsafi Z Khan M Sohrabi C. Impact of the coronavirus (COVID-19) pandemic on surgical practice-Part 2 (surgical prioritization). Int J Surg. (2020) 79:233–48. 10.1016/j.ijsu.2020.05.002 32413502 PMC7217115

[19] Turk MA McDermott S. The COVID-19 pandemic and people with disability. Disability Health J. (2020) 13:100944. 10.1016/j.dhjo.2020.100944 32475803 PMC7254018

[20] Koon LM Greiman L Schulz JA Goddard KS Nzuki IM Hall JP. Examining the effects of the COVID-19 pandemic on community engagement for people with mobility disabilities. Disabil Health J. (2022) 15:101212. 10.1016/j.dhjo.2021.101212 34531174 PMC8418869

[21] Izutsu T. Disability-inclusive disaster risk reduction and humanitarian action: An urgent global imperative. Available online at: https://www.un.org/development/desa/disabilities/wp-content/uploads/sites/15/2020/03/Final-Disability-inclusive-disaster.pdf (accessed November 29, 2019).

[22] Pineda VS Corburn J. Disability, urban health equity, and the coronavirus pandemic: promoting cities for all. Erratum in: *J Urban Health*. (2021) 98:308. 10.1007/s11524-020-00490-2 33649907 PMC7919625

[23] Krahn GL Walker DK Correa-De-Araujo R. Persons with disabilities as an unrecognized health disparity population. Am J Public Health. (2015) 105:S198-206. 10.2105/AJPH.2014.302182 25689212 PMC4355692

[24] Sorinmade OA Kossoff L Peisah C. COVID-19 and telehealth in older adult psychiatry: Opportunities for now and the future. Int J Geriatr Psychiatry. (2020) 35:1427–30. 10.1002/gps.5383 32729632

[25] Brooks B Hay A. Hoarding in the USA? Coronavirus Sparks Consumer Concerns. Available online at: https://www.reuters.com/article/us-china-health-usa-hoarding/hoarding-in-the-usa-coronavirus-sparks-consumer-concerns-idUSKCN20M37V (accessed February 28, 2020).

[26] Campbell VA Gilyard JA Sinclair L Sternberg T Kailes JI. Preparing for and responding to pandemic influenza: Implications for people with disabilities. Am J Public Health. (2009) 99:S294-300. 10.2105/AJPH.2009.162677 19797741 PMC4504380

